# Reply to Dubbert and von Bünau, “A Probiotic Friend”

**DOI:** 10.1128/msphere.00906-21

**Published:** 2021-12-22

**Authors:** Jean-Philippe Nougayrède, Eric Oswald

**Affiliations:** a IRSD, INSERM, INRAE, Université de Toulouse, ENVT, Toulouse, France

## REPLY

We read with great interest the comment letter to the editor by Dubbert and von Bünau (1) from Ardeypharm regarding our work, recently published in *mSphere*, on the production of the genotoxin colibactin by the Nissle 1917 probiotic (2). We fully understand and acknowledge the strong commitment made by Ardeypharm—which markets Nissle 1917 under the trade name Mutaflor—with regard to the safety of its customers and the improvement of patient health.

In their letter (1), the authors question our results on the *in vitro* and *in vivo* genotoxicity of Nissle 1917 (2) with respect to their previous publication in which they stated that this E. coli strain did not exert such genotoxicity in standard tests (3). These standard tests, which are recommended by the FDA and the OECD, are suitable for testing the mutagenic effect of chemicals on human health. However, we continue to believe that the tests used by the authors are not the most suitable for testing the mutagenic effect of the Nissle 1917 bacteria. The first test they used is an Ames mutation reversion test in Salmonella, exposed to a supernatant of Nissle 1917 (3). However, no genotoxic activity associated with colibactin could be observed in the supernatant (4). It is also well-known that Salmonella is killed by the siderophore microcins produced by Nissle 1917 (5). These microcins are responsible for the antagonistic activity of Nissle 1917 at the heart of its probiotic property, which is linked to the production of colibactin (6, 7). Although Dubbert and von Bünau write in their letter that all the appropriate controls were performed and that bacterial lawns were observed on the control plates, no quantitative data pertaining to these important controls is mentioned in the publication. The reader therefore has no way of assessing whether the microcin activity of Nissle 1917 generated a false-negative result or not. We agree with the authors that it is important to also test the contact-dependent mutagenic activity of Nissle 1917. Dubbert et al. used a second modified Ames test in which an antibiotic was added so as to inhibit the growth of Nissle 1917, but not that of Salmonella (3). However, it is known that inhibiting growth with an antibiotic impairs the production of colibactin (4). Without using a purified and stable form of colibactin, it is impossible to use the Ames test. We therefore maintain that these Ames tests could not work to test the mutagenicity of Nissle 1917 bacteria. Similarly, the Ames test could not demonstrate the mutagenicity of Helicobacter pylori (8), which is a well-recognized oncomicrobe.

We agree with the authors that it is crucial to test *in vivo* the genotoxicity of Nissle 1917. We observed histone H2AX phosphorylation (in response to DNA damage) in 8-day-old mice or in axenic mice which had been monocolonized with Nissle 1917, but not with a mutant unable to produce colibactin (2), which is similar to the results reported in young rats or monoxenic mice colonized with other *pks^+^* E. coli strains (9, 10). Dubbert et al. reported a contradictory result in another Nissle 1917 genotoxicity test in rats where no detectable DNA damage was demonstrated by a standard comet assay (3). This comet assay allows for the detection of DNA breaks inflicted by colibactin at very high doses, as observed in cells exposed to a very high number of *pks^+^* E. coli (4). We now know that these lesions are derived from the generation of DNA interstand cross-links (ICLs) by colibactin, which can break by way of depurination and, consequently, generate DNA breaks (11, 12). However, at a more moderate dose of colibactin, ICL lesions remain in the majority, and the comet assay remains negative because ICLs inhibit DNA electrophoretic migration (13). Accordingly, it is quite possible that the negative result the authors obtained arises from the low sensitivity of the comet assay to colibactin-induced DNA damage at a realistic dose *in vivo*.

We agree with the authors that murine tests are not an adequate representation of the use of Nissle 1917 in humans, which exhibit in particular normal or, on the contrary, dysbiotic microbiota and altered barrier functions in inflammatory bowel disease patients. However, the recent detection of the colibactin mutational signature in colorectal tumor banks indicates that colibactin produced by *pks^+^* E. coli is indeed produced, and genotoxic, in humans (14, 15). We agree with Dubbert and von Bünau who stated in their letter (1) that these *pks^+^* E. coli bacteria are frequently found in young children. Previous work has shown that *pks^+^* E. coli induces DNA damage during the perinatal colonization phase in a rat model, with persistent changes to the intestinal tissue homeostasis and cell turnover (10). In addition, the mutational signature of colibactin has also been observed in the intestinal crypts of healthy young humans (16), suggesting that colibactin can imprint damage during childhood, thus leading to potential long-term consequences (17).

The detection of the colibactin mutational signature in colorectal tumors, the understanding of the mechanism of DNA damage, mutagenesis, and transformation, as well as various epidemiological studies showing more E. coli *pks*^+^ in colorectal cancer (CRC) patients and finally the experimental reproduction of colibactin-aggravated CRC in mouse models all concur to implicate colibactin in this type of cancer (18). However, we agree with Dubbert and von Bünau (1) that CRC is a multifactorial disease and that other risk factors—in particular, diet—are very important. The authors submit that the mutational signature of colibactin is found in only 5% of the analyzed tissues. Indeed, this signature was identified in 5.3% of the mutations in the adenomatous polyposis coli (APC) gene, mutated in colorectal tumors (14). However, care should be taken not to minimize and misinterpret this figure. First, the colibactin-specific mutation in the APC gene suggests a causal role in CRC. Second, although a significant fraction of driver mutations is induced by other mutagenic processes (including endogenous), a tumor typically harbors between 2 and 10 driver mutations, which can be induced by various environmental genotoxins such as colibactin. This may be sufficient to induce full malignancy (19). The colibactin mutational signature is found in genes other than APC, and another study identifies it in nearly 10% of CRCs (15). In addition, colibactin has been shown to also induce large-scale DNA damage and chromosome instability, which can result in nonspecific mutations and cell transformation (18, 20, 21). Consequently, colibactin may be responsible for inducing a larger fraction of cancers than the fraction of mutations attributed to colibactin in the APC gene.

We again fully agree with Dubbert and von Bünau (1) that CRC is multifactorial, diet and intestinal inflammation contributing in a major way. Indeed, we have shown that food contaminants may participate in conjunction with colibactin to the mutagenesis process (22). As observed by the authors, intestinal inflammation is a well-known factor in CRC, and promotes colibactin expression by *pks^+^* E. coli and tumorigenesis (9, 23). It is clear that Nissle 1917 has interesting properties with regard to reducing intestinal inflammation, but to the best of our knowledge, no study has yet thoroughly examined whether its use was associated with a decreased or increased risk of developing colorectal cancer. On the other hand, certain molecules with real potential—such as mesalamine—decrease both inflammation and the production of colibactin by *pks^+^* E. coli, and therefore reduce the risk of CRC (24, 25).

In conclusion, the impact of colibactin in cancer can still be discussed and needs to be further studied. However, in light of our results (2) and of the arguments presented above, it would seem sensible to reevaluate the use of colibactin-producing E. coli for therapeutic use in humans.
